# The Spread of the COVID-19 Outbreak in Brazil: An Overview by Kohonen Self-Organizing Map Networks

**DOI:** 10.3390/medicina57030235

**Published:** 2021-03-03

**Authors:** Diego Galvan, Luciane Effting, Hágata Cremasco, Carlos Adam Conte-Junior

**Affiliations:** 1 COVID-19 Research Group, Center for Food Analysis (NAL), Technological Development Support Laboratory (LADETEC), Cidade Universitária, Rio de Janeiro 21941-598, RJ, Brazil; conte@iq.ufrj.br; 2 Laboratory of Advanced Analysis in Biochemistry and Molecular Biology (LAABBM), Department of Biochemistry, Federal University of Rio de Janeiro (UFRJ), Cidade Universitária, Rio de Janeiro 21941-909, RJ, Brazil; 3 Nanotechnology Network, Carlos Chagas Filho Research Support Foundation of the State of Rio de Janeiro (FAPERJ), Rio de Janeiro 20020-000, RJ, Brazil; 4 Chemistry Department, State University of Londrina (UEL), Londrina 86057-970, PR, Brazil; luciane.effting@uel.br (L.E.); hagata@uel.br (H.C.)

**Keywords:** coronavirus disease, unsupervised neural networks, SARS-CoV-2, tropical country, Latin America, Kohonen map

## Abstract

*Background and objective*: In the current pandemic scenario, data mining tools are fundamental to evaluate the measures adopted to contain the spread of COVID-19. In this study, unsupervised neural networks of the Self-Organizing Maps (SOM) type were used to assess the spatial and temporal spread of COVID-19 in Brazil, according to the number of cases and deaths in regions, states, and cities. *Materials and methods*: The SOM applied in this context does not evaluate which measures applied have helped contain the spread of the disease, but these datasets represent the repercussions of the country’s measures, which were implemented to contain the virus’ spread. *Results:* This approach demonstrated that the spread of the disease in Brazil does not have a standard behavior, changing according to the region, state, or city. The analyses showed that cities and states in the north and northeast regions of the country were the most affected by the disease, with the highest number of cases and deaths registered per 100,000 inhabitants. *Conclusions*: The SOM clustering was able to spatially group cities, states, and regions according to their coronavirus cases, with similar behavior. Thus, it is possible to benefit from the use of similar strategies to deal with the virus’ spread in these cities, states, and regions.

## 1. Introduction

Since the first reports of novel pneumonia caused by a virus called “coronavirus” (COVID-19), in December 2019 in Wuhan, Hubei province in China, the disease has spread worldwide quickly [1,2]. COVID-19 is a highly transmittable viral infection caused by severe acute respiratory syndrome coronavirus 2 (SARS-CoV-2), being declared a pandemic by the World Health Organization (WHO) on 11 March 2020 [3]. Currently, SARS-CoV-2 infections have more than 67 million cases, with more than 1.5 million deaths by December 2020 worldwide [4].

The WHO information indicates that currently, the epicenter of the novel coronavirus in the world is in the south of the planet, especially in Brazil and other Latin American countries. Nowadays, Brazil is the third country with the highest number of confirmed cases and deaths, with more than 10 million cases and 240,000 deaths, behind only the United States and India [4].

The main frontline in the fight against the pandemic is the relentless pursuit for the development of vaccines and medicines aimed for immunization and treatment of the problems caused by COVID-19 [5,6]. Other measures adopted, such as lockdown, seek to reduce the disease’s spread [7]. However, this type of measure may not have the same effect in different countries and regions; many factors can interfere in these measures’ efficiency, as climatic, economic, and cultural differences [8,9].

In this context, computational tools for data analysis can contribute to decision-making regarding prevention measures for each case [10]. Artificial intelligence (AI) has already been used to model and simulate the spread and lethality of COVID-19 in some countries [11,12,13]. Data mining is now essential due to the massive amount of data and information generated daily by health and government agencies [14,15]. Among the several types of artificial neural networks (ANNs), there are the Self-Organizing Maps (SOM) or Kohonen Map. In this type of ANN, the intrinsic relationships among samples or variables in a dataset are represented by a competitive and unsupervised learning method [16,17]. SOM is a method for analyzing multivariate data used for exploratory and clustering problems, which may be considered a non-linear generalization of principal component analysis (PCA). This method follows less conventional statistical principles, without the need for in-depth knowledge in statistics and multivariate analysis [10,18].

The SOM algorithm consists of input nodes and a grid of computational connected nodes (neurons), which compete among themselves for activation as the one that most closely resembles the input vector [16]. If the input data exhibit some similarity across the input classes, the neurons will organize themselves, showing similarity patterns in a grid, usually a two-dimensional rectangular or hexagonal grid [10]. The data mapping in the array of neurons preserves topological relationships and the probability of distribution. Therefore, the Kohonen Map is a powerful tool for interpreting data [19].

A perspective on the use of Machine Learning (ML), more broadly, AI to deal with many aspects of the crisis caused by COVID-19, can be consulted in the review of Bullock and collaborators [20], which demonstrate simple approaches, such as the unsupervised k-means algorithm, until more sophisticated ones that use the latent features of autoencoders, trained initially, to predict infection rates, to identify similar groups of regions or countries, applying the modified autoencoder (MAE) and topological autoencoder (TA) algorithms.

The TA is a novel topological neural network for similarity maps and pairwise predictions that was initially proposed by Hartono [21]. This algorithm was applied to evaluate the transmission dynamics of COVID-19 for more than 250 countries and territories. The TA algorithm produces a two-dimensional map, which projects the topological structures of these data dynamics in a similar way to SOM, but it is more flexible, not only to visualize but also to predict COVID-19 cases globally. This obtained map allows researchers to define a target country to be predicted, which is then used to locate a reference country with similar dynamics that started earlier; then, the map uses the reference dynamics to train a long short-term memory (LSTM) for a prediction task.

The SOM clustering skills were assessed by Melin et al. [14] to spatially group Mexico countries and states, according to their coronavirus cases. Galvan et al. [10] verified the ability to group SOM using socioeconomic, health, and safety data to verify the relationship between the number of cases and deaths by COVID-19 in Brazilian states. In this study, we propose to evaluate and map the spatial and temporal spread of COVID-19 in Brazil by regions, states, and cities, according to the number of cases and deaths by a particular form of unsupervised neural networks of the Self-Organizing Maps (SOM) type.

## 2. Methodology

### 2.1. Database

The dataset used in neural networks was obtained on 31 May 2020, from the Ministério da Saúde do Brasil—MS (Ministry of Health of Brazil), the government sector responsible for administering public health in the country. The website is updated daily, and all information about the pandemic in the country is in the public domain, following current legislation [22].

Novel and cumulative numbers of cases and deaths from COVID-19 were analyzed for ten epidemiological weeks. According to the international convention, each week starts on Sunday and ends on Saturday. The period studied was from 28 March to 30 May 2020 (93 days), corresponding to the period from weeks 13 to 22 of the year. The “novel” symbology represents the numbers of cases and deaths recorded in each of the ten epidemiological weeks evaluated, while the term “cumulative” represents the sum of the numbers of cases and deaths recorded during the previous epidemiological weeks.

For spatial and temporal analysis, input data from the SOM network were used since the first case of COVID-19 in Brazil, registered on 26 February 2020 in São Paulo. From that date, the numbers of novel cases and deaths were added to the dataset until 30 May periods that represent epidemiological weeks from 9 to 22. For the temporal analysis, the input data represent the number of novel cases and deaths registered by epidemiological weeks between 9 and 22 but are shown as 13 to 22 on the charts. It is noteworthy that the epidemiological data for weeks 9 to 12 were computed as accumulated and added to week 13, following the criteria adopted by government agencies that provided data on disseminating diseases, that is, from the epidemiological week 13.

Three different approaches were carried out to analyze the novel and cumulative numbers of cases and deaths from COVID-19, one for cardinal location (regions), one for federative units (states), and one for the most populous cities in Brazil. The regions representative data show the sum of the states’ cases of those regions. Meanwhile, the states’ data represents the sum of the cities’ cases belonging to these states.

In the study of Brazilian cities, all capitals of each federative unit were chosen, 26 cities plus Brasília, the federal capital. Another 25 cities (not capitals), with more than 500,000 inhabitants, were included in the study, totaling 52 of the 5570 cities in Brazil, representing 32% of the Brazilian population and corresponding to 53% and 65% of cases and deaths by COVID-19, respectively, until the epidemiological week 22.

All data were expressed by the number of cases per 100,000 inhabitants, which were converted by the data of the Brazilian population estimated in 2019 by the Instituto Brasileiro de Geografia e Estatística—IBGE (Brazilian Institute of Geography and Statistics), the public agency responsible for conducting censuses and organizing information related to the country’s geosciences and social, demographic and economic statistics [23].

Table 1 shows Brazil’s divisions by regions, states, most populous cities, and their populations. The regions and states’ populations represent a share of the total Brazilian population estimated at 210,147,125 people, while each city’s share represents part of each state’s total population.

The percentages on the right to each region and state are considered over the total Brazilian population. For example, 18,430,980 inhabitants in the north region correspond to 9% of the Brazilian population, or 45,919,049 inhabitants in São Paulo state correspond to 21.9% of the Brazilian population. The percentages on the right of the cities refer to the total population of the state which they belong. For example, the 612,547 inhabitants of Cuiabá represent 17.6% of the Mato Grosso do Sul state.

### 2.2. Proposed Method

The proposal was to evaluate the spatial and temporal spread of COVID-19 cases in Brazil, using a particular type of unsupervised artificial neural networks (ANN) called Self-Organizing Map (SOM) or Kohonen Map [17,24].

SOM is a method for analyzing multivariate data used for exploratory and clustering problems [18]. The Kohonen algorithm consists of input nodes and a grid of connected computational nodes (neurons), which compete among themselves for activation as the one that most closely resembles the input vector. This algorithm begins by initializing the first grid with random synaptic weights, and no organization is applied to the map. Three key processes take place: competition, cooperation, and synaptic adaptation [17,24].

The SOM routine developed was used according to the algorithm described in Haykin [16]. The function is chosen to represent the topological neighborhood in Equation (1).

(1) $$h_{j,i}=\exp\left(-d_{j,i}^{2}/2\sigma^{2}\right)$$

where *σ* is the effective radius of the topological neighborhood, and $d_{j,i}$ is the lateral distance between the “winning neuron” *i* and the excited neuron *j*. The “winning neuron” is the particular neuron that best matches the input vector **x**, summarizing the competitive process’ essence between neurons. Over the training epochs, there is a reduction in the neighborhood’s size due to exponential decay, as shown in Equation (2).

(2) $$\sigma \left(n\right)=\;\sigma_{0}\exp\left(-n/\tau_{1}\right)\;\;\;n=0,1,2,\ldots$$

where *σ*_0_ is the effective radius in the algorithm initialization, *τ*_1_ is the time constant, with *τ*_1_ = 1000/log *σ*_0_ being recommended, and *n* is the number of training epochs.

During the adaptive process for the self-organized formation of a feature map, the synaptic weight vector (**w***_j_*) of the *j* neuron in the grid must be modified in relation to the input vector **x,** where **x** is an input vector randomly selected from the input spaces. The modification process is a modification of the Hebb postulate of learning, described by Equation (3).

(3) $$w_{j}\left(n+1\right)=\;w_{j}\left(n\right)+\eta \left(n\right)h_{j,i\left(x\right)}\left(n\right)\left(x-w_{j}\left(n\right)\right)$$

where *η*(*n*) is the learning rate, which is variable and decreases during the training epochs, *n*. The learning rate decrease may be modeled by an exponential decay, as described in Equation (4). In this equation, *η*_0_ is the initial learning rate, and *τ*_2_ is another time constant; the recommended values are 0.1 and 1000, respectively:

(4) $$\eta \left(n\right)=\;n_{0}\exp\left(-n/\tau_{2}\right).$$

At the end of the learning process, each sample is associated with its winning neuron, forming a topological map that allows clusters’ visualization and checks the neighborhood relationship between the groups formed. It is also possible to visualize the results by the weight maps or weight plan through a level contour graph, representing each variable’s influence for the sample segmentation. Together with the topological map, they observe behavior rules for each group formed and infer each variable’s influence on the result obtained [25]. For the weight maps build, the **w***_j_* values for each variable were interpolated by “nearest” and “spline” functions (in Matlab) to regularize the hexagonal grid, using the automatic arrangement of the samples obtained from the topological map.

The following variables were provided as input variables for the SOM: novel and accumulated numbers of cases and deaths by COVID-19 per 100,000 inhabitants, as shown in Table 2. The dataset generated six analysis groups (three spatial and three temporal), totaling 3488 data used as input variables for each SOM network, as presented in the previous section. The SOM setup was a hexagonal topology of 3 × 3 to 25 × 25 neurons in each dimension were tested. The map was trained 7000 epochs to ensure the convergence of the mean quantization error (MQE).

### 2.3. Computer Processing and Program

We used a computer Intel^®^ Core™ i7–4790 CPU^©^ 3.60 GHz, 32 GB RAM, and 250 GB HDD. The neural network routine developed was applied according to the algorithm described by Haykin [16] and was processed in the software Matlab^®^ (MathWorks, Natick, MA, USA).

## 3. Results

**Figure 1:**
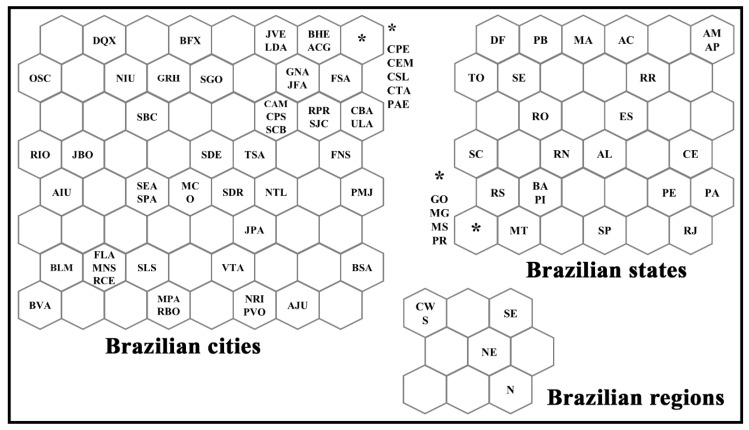
Topological maps for the distribution of Brazilian cities, states, and regions according to the winning neuron. See abbreviations in Table 1. Where: * represents the clusters of cities and states in each network grouped in the same winning neuron.

In the topological map, each Brazilian city, state, or region is associated with a respective winning neuron, that is, that neuron that best represents it in the network for cities, states, or regions, see Figure 1. The SOM classifies the input data as clusters that can be formed by one or more neurons. The definition of clusters is characterized by the presence of empty neurons among the groups. Nearby clusters share some similarity—that is, the greater the Euclidean distance, the greater the behavior difference [10].

Figure 1 shows the topological maps generated after the training of each SOM network. The random distribution of Brazilian cities, states, and regions on the maps indicates that the numbers of novel and accumulated cases and deaths by COVID-19 change according to the country’s geographic location. At first, the analysis may seem trivial, although the SOM algorithm application does not allow the possibility of making trivial assumptions that simple visual analyses allow. Thus, visualizing and understanding how the virus is transmitted and its likely effect on various demographic data and geographic locations are crucial for public health interventions [20].

An overview of Figure 1 allows us to state clusters between cities and states belonging to the same region. Using the cluster in the upper right corner of the network for Brazilian cities as an example, we can verify that most of these cities belong to the states of the Central-West (CW), Southeast (SE), and South (S) Brazilian regions. We can also verify, in the network representing the states, the formation of a cluster in the lower left quadrant of the map. In this cluster are present most of the Brazilian states that belong to the Central-West (CW), Southeast (SE), and South (S) regions of the country, which are represented mainly by the states of Goiás (GO), Minas Gerais (MG), Mato Grosso do Sul (MS), Paraná (PR), Rio Grande do Sul (RS), and Mato Grosso (MS). It is also possible to verify the formation of a cluster in the upper left quadrant of the map represented by the South (S) and Central-West (CW) regions.

### 3.1. Spread of COVID-19 by Brazilian Regions

The first dataset for the five Brazilian regions represents the sum of the number of cases and deaths accumulated since the first COVID-19 record in Brazil until week 22. Moreover, the numbers of novel cases and deaths are equivalent to the sum of cases registered between weeks 13 and 22.

**Figure 2:**
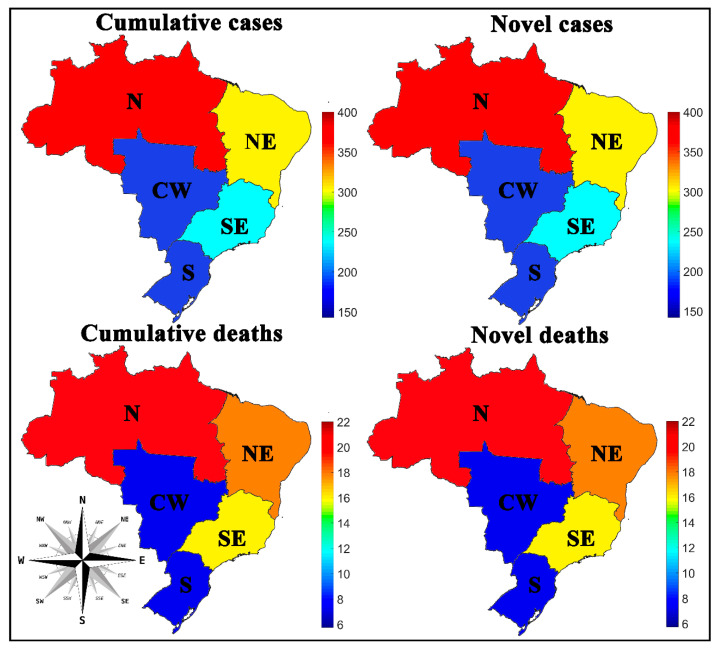
Spatial aspect of the coronavirus 2019 (COVID-19) spread in Brazilian regions per 100,000 inhabitants. N (North), NE (Northeast), SE (Southeast), S (South), and CW (Central-West).

**Figure 3:**
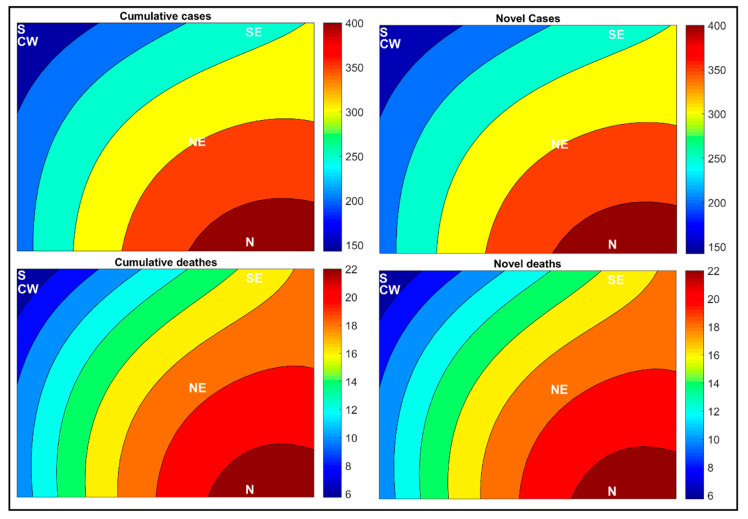
Weight maps for the spatial aspect of the COVID-19 spread in Brazilian regions per 100,000 inhabitants. N (North), NE (Northeast), SE (Southeast), S (South) and CW (Central-West).

Figure 2 shows a spatial aspect of the COVID-19 spread by Brazilian regions, while the values observed for the input variables are indicated through numerical color scales, per 100,000 inhabitants. For a better representation, the values collected after the SOM analysis for Brazilian regions were transposed through the color scale to the Brazilian cartographic map of each variable. The original SOM outputs of the weight maps for the variables are shown in Figure 3.

In Figure 2 and Figure 3, it is important to note that the novel and accumulated numbers of cases and deaths by COVID-19, per 100,000 inhabitants, exhibit visual similarities between the left and right side, because the period studied represents the exponential phase of COVID-19 cases in the country since the period before week 13 has a low number of registered cases. During this period, these data represent part of the first wave’s peak in numbers of cases and deaths, which caused this similar behavior. This same explanation serves as the basis for other networks representing the states and cities distribution.

**Figure 4:**
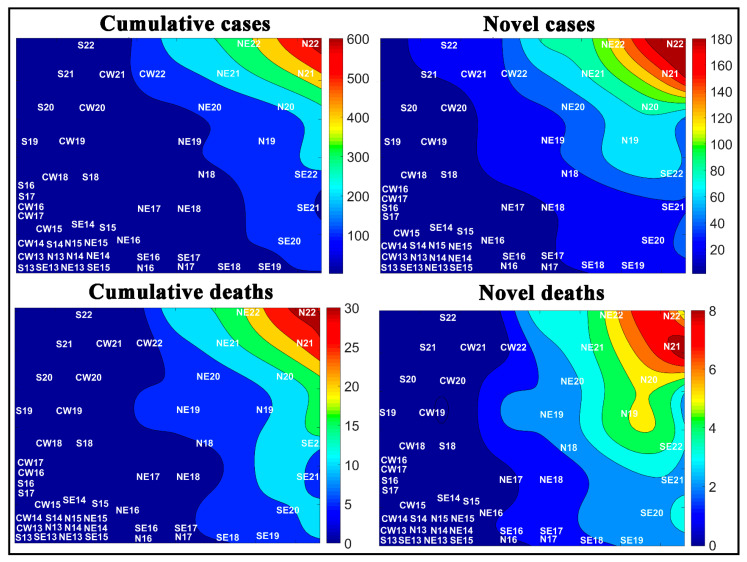
Temporal aspect of the COVID-19 spread in Brazilian regions per 100,000 inhabitants. Where the letters represent the five regions, and the numbers represent the epidemiological weeks. N (North), NE (Northeast), SE (Southeast), S (South), and CW (Central-West).

In Figure 4, the SOM segmented the epidemiological weeks according to the COVID-19 propagation time in an almost identical manner. The interpretation of the disease spread is easily understood in the weight maps. In the first pandemic epidemiological weeks, all Brazilian regions had lower rates of cases and deaths due to COVID-19, which were represented in the lower left quadrant of the maps. After a few epidemiological weeks, some Brazilian regions stood out considering the numbers of cases and deaths by COVID-19. Figure 4 shows that the highest rates are equivalent to epidemiological weeks 22, 21, and 20 in the North (N22, N21, and N20), followed by the Northeast (NE22 and NE21), which are found on the upper right side of the weight maps, indicating that there is an increase in the number of cases and deaths by novel and accumulated COVID-19 in Brazil as a whole, i.e., the country has not still reached a plateau in the cases’ growth.

### 3.2. Spread of COVID-19 by Brazilian States

The second dataset for the Brazilian states represents the sum of the number of cases and deaths accumulated since the first COVID-19 record in Brazil until week 22. The numbers of novel cases and deaths are equivalent to the sum of cases registered between weeks 13 and 22 for all states.

**Figure 5:**
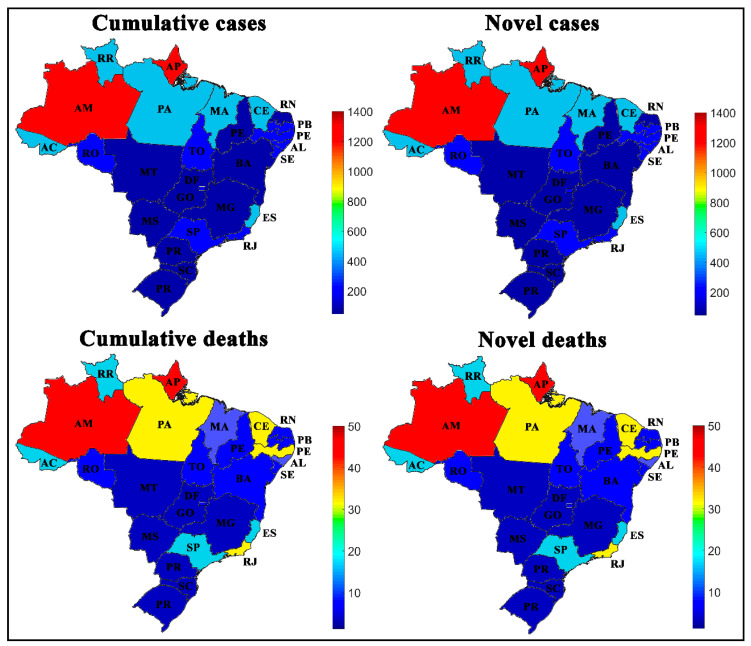
Spatial aspect of the COVID-19 spread in Brazilian states per 100,000 inhabitants. See state abbreviations in Table 1.

**Figure 6:**
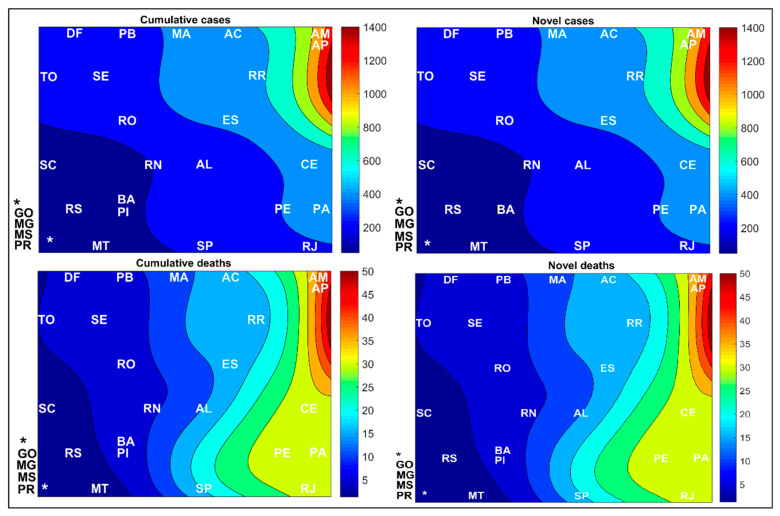
Weight maps for the spatial aspect of the COVID-19 spread in Brazilian states per 100,000 inhabitants. See state abbreviations in Table 1.

Figure 5 demonstrates that all states in the South and Central-West regions had the lowest rates of novel and accumulated cases and deaths by COVID-19. The states of Amazonas (AM) and Amapá (AP) had a novel and accumulated number of cases and deaths per 100,000 inhabitants that was higher than all other states, while the most populous states such as São Paulo (SP) and Rio de Janeiro (RJ) had an average number of cases. For a better representation, the values collected after the SOM analysis for Brazilian regions were transposed through the color scale to the Brazilian cartographic map of each variable. The original SOM outputs of the variables weight maps are shown in Figure 6.

**Figure 7:**
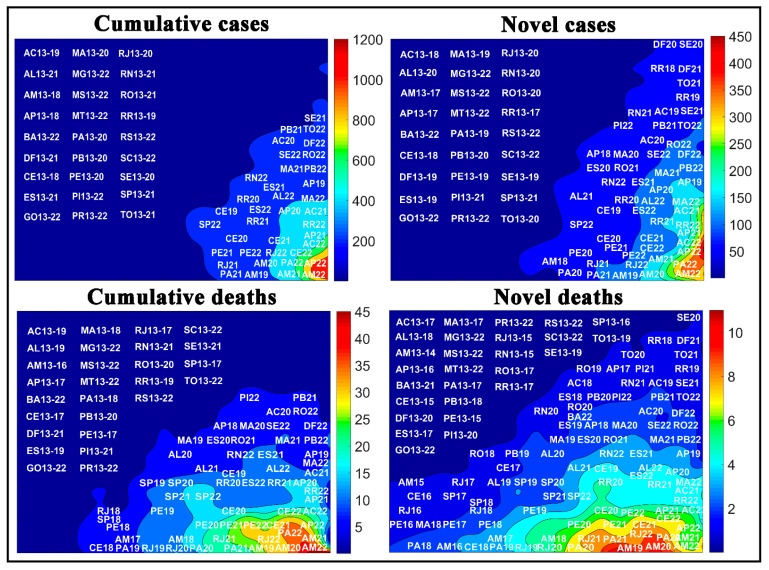
Temporal aspect of the COVID-19 spread in Brazilian states per 100,000 inhabitants, where the letters represent the states and the numbers represent the epidemiological weeks. See state abbreviations in Table 1.

Another approach for analyzing the novel and accumulated case and death rates for COVID-19 was to assess the temporal aspect of the COVID-19. Figure 7 shows the numbers of novel and cumulative cases and deaths by COVID-19 in Brazilian states in each epidemiological week. According to the weight maps, the largest number of cases per 100,000 inhabitants.

### 3.3. Spread of COVID-19 by Brazilian Cities

The third dataset for the Brazilian cities represents the sum of the number of cases and deaths accumulated until week 22 for the 52 most populous cities of the country. The numbers of novel cases and deaths are equivalent to the sum of cases registered between weeks 13 and 22 for the chosen cities.

**Figure 8:**
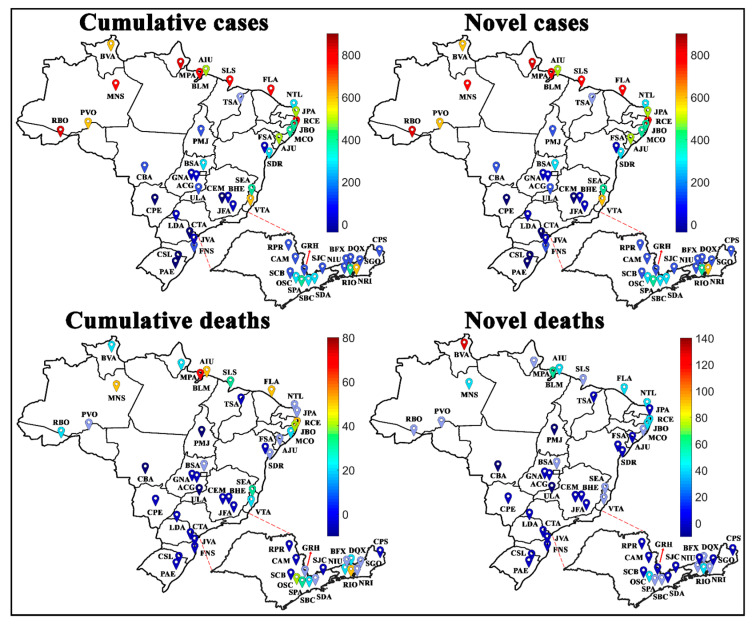
Spatial aspect of the COVID-19 spread in Brazilian cities per 100,000 inhabitants. For better viewing, São Paulo and Rio de Janeiro states are zoomed. See city abbreviations in Table 1.

**Figure 9:**
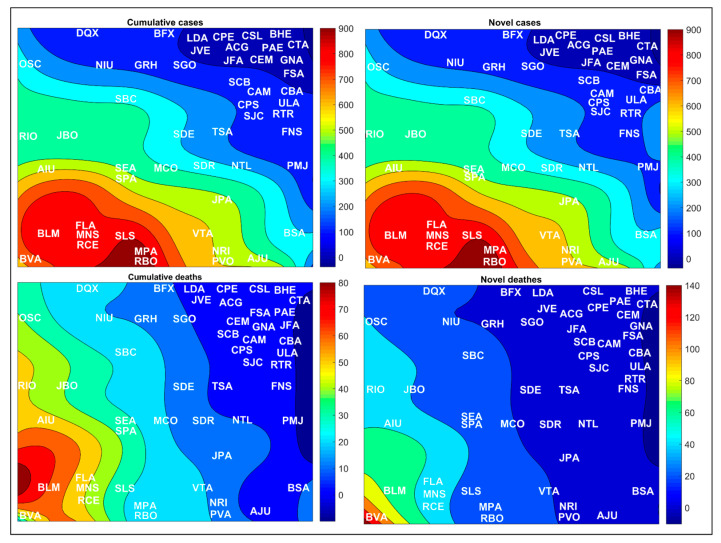
Weight maps for the spatial aspect of the COVID-19 spread in Brazilian cities per 100,000 inhabitants. For better viewing, São Paulo and Rio de Janeiro states are zoomed. See city abbreviations in Table 1.

Figure 8 shows the variation in COVID-19 case rates for cities in Brazil; in general, the cities located in the South and Central-West regions of Brazil have the lowest reported rates. Meanwhile, the cities most affected by the SARS-CoV-2, with the highest numbers of novel and accumulated cases were the cities located in the North and Northeast regions of Brazil, with emphasis on Belém (BLM), Macapá (MPA), Manaus (MNS), Rio Branco (RBO), Fortaleza (FLA), Recife (RCE), and São Luís (SLS). Belém (BLM) had the highest number of accumulated deaths, while Boa Vista (BVA) had the highest number of novel deaths recorded per 100,000 inhabitants. For a better representation, the values collected after the SOM analysis for Brazilian regions were transposed through the color scale to the Brazilian cartographic map of each variable. The original SOM outputs of the variables weight maps are shown in Figure 9.

**Figure 10:**
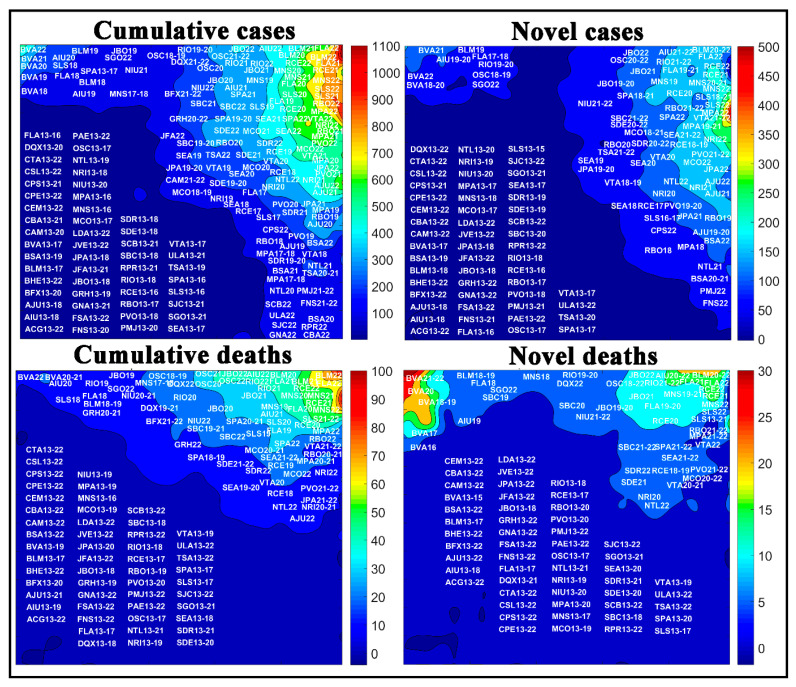
Temporal aspect of the COVID-19 spread in Brazilian cities per 100,000 inhabitants, where the letters represent the cities, and the numbers represent the epidemiological weeks. See city abbreviations in Table 1 and the amplified images in the Supplementary Materials (Figures S1–S4).

Another approach for analyzing the novel and accumulated case and death rates for COVID-19 was to assess the temporal aspect of the COVID-19. Figure 10 shows the numbers of novel and cumulative cases and deaths by COVID-19 in Brazilian cities in each epidemiological week. According to the weight maps, the largest number of cases per 100,000 inhabitants. For a better view of each window in Figure 10, the reader can consult Figures S1–S4 of the Supplementary Materials.

## 4. Discussion

In the current world scenario, many governmental and non-governmental institutions offer a lot of information about the coronavirus pandemic. However, only a qualitative analysis of these data can mask relevant information or even generate erroneous conclusions. Today, computer screening is one of the main focuses for the solution and evaluation of the measures adopted to contain the pandemic [13,14].

Data analysis tools are indispensable in this context. There are many available, from conventional statistical methods to more sophisticated tools. SOM stands out among the options, which has a good performance in recognizing and classifying patterns [10,18]. Previous studies have compared the SOM algorithm’s performance to another unsupervised clustering method, such as the Hierarchical Cluster Analysis (HCA) method applied to the numbers of cases and deaths by COVID-19. According to the analyses, the results obtained between SOM and HCA were very similar. More details about comparing methods can be seen in Galvan’s work [10]. Recently, Hartono [21] and Hu et al. [26] went beyond topological visualization and clustering skills. In their studies, the authors assessed the ability to predict COVID-19 cases with approaches similar to SOM, using more sophisticated ones as TA-LSTM and MAE-k-means, to evaluate the transmission dynamics of COVID-19 in countries, provinces, cities, and regions.

SOM is considered a special class of neural network grids. These grids are based on competitive learning, in which the neurons leaving the grid to compete with each other to be activated or triggered. An outgoing neuron that wins the competition is called a “winner neuron”. Thus, the neurons’ location becomes ordered with each other so that a coordinate system for different input characteristics is created on the grid, which is characterized by the formation of the topological map, according to the input patterns or according to the intrinsic statistical characteristics contained in these input patterns [16].

In the network representing the distribution of the most populous Brazilian cities, states and regions, we can see the formation of some clusters among them (see Figure 1). These clusters indicate that these cities, states, or regions have similarities in the number of cases and deaths by COVID-19. As an example, we have the cluster formed in the upper right corner of the network for cities, represented by Campo Grande (CPE), Florianópolis (FSA), Belo Horizonte (BHE), among other cities present in this quadrant. We can say that these Brazilian cities show similar behavior in numbers of cases and deaths by COVID-19.

Another typical analysis that can be made is the neighborhood relationship, which indicates how similar these clusters are. The presence of empty neurons between these clusters increases the distance, causing dissimilarity between them. For example, the cluster formed by the cities in the upper right corner of the map differs more from the cluster in the lower left corner of the map than the upper left cluster. This same observation and interpretation can be made for the other clusters in this network, being valid for the other networks that represent Brazilian states and regions.

In general, topological maps allow us to verify which Brazilian cities, states, or regions follow similar behaviors or not in the number of cases and deaths by COVID-19, using a pattern recognition method. According to Melin et al. [14], SOM’s clustering skills allow us to spatially group countries or states similar to their coronavirus data. Thus, locations with similarities can benefit from using analogous strategies to deal with the virus’ spread. In addition, topological maps allow the extraction of resources that can be used for the prediction task [21,26].

Only topological maps do not allow us to state which Brazilian cities, states, and regions have had the highest or lowest incidence of cases and deaths due to COVID-19. For this purpose, weight maps will be used in future discussions. The weight maps represent the topological maps’ overlapping on the segmentation of Brazilian cities, states, and regions. In the weight maps, the values observed for the input variables are indicated by the color scale. For a better representation, the values collected after the SOM analysis were transposed through the color scale to the Brazilian cartographic map of each variable, which allows a vertical interpretation determined by the cases per 100,000 inhabitants. This procedure was adopted to facilitate the interpretation of readers unfamiliar with the Kohonen map. However, as a disadvantage, the transposition of the SOM network’s original outputs suppresses the neighborhood relationship. That is, it does not allow us to verify how similar or not these clusters are.

The weight maps can be seen as a horizontal representation of the dataset; they do not directly correlate with the geographic location or the numbers of epidemiological weeks, since SOM is an unsupervised data analysis method. The weight maps represent the topological maps overlap for each variable and follow the same interpretation line described in the topological maps. In other words, they follow a clustering principle but demonstrate the importance of each variable by weights, which is represented by the color scale.

### 4.1. Spread of COVID-19 by Brazilian Regions

The results show that the North (N) and Northeast (NE) regions have the highest rates of novel and accumulated cases and deaths by COVID-19 per 100,000 inhabitants, which are followed by the Southeast region (SE), the most populous region in the country, and then the South (S) and Central-West (CW) regions, which had the lowest rates. Reports indicate that Brazil’s highest number of COVID-19 cases is in the Southeast region, although the SOM network has shown that the highest rates per 100,000 inhabitants are in the North and Northeast regions of the country, see Figure 2 and Figure 3.

In Figure 3, the weight maps indicated that the cluster formed by the Central-West (CW) and South (S) regions of the country showed a similarity because they had lower numbers of novel and accumulated numbers of cases and deaths. Regions with higher incidences, such as the North (N) and Northeast (NE), were more distant, and the Southeast (SE) had intermediate values. We emphasize that the SOM applied in this context does not allow us to assess which measures have been applied to contain the disease’s spread in Brazil. However, these data represent the repercussion of the measures effects used in each Brazilian region, state, or city, which were adopted to contain the spread of infections caused by the virus. In other works published by our group, we discussed possible factors that may influence the virus’ spread, such as socioeconomic, political, health, safety [10,27], and weather [28].

Another approach for analyzing the novel and accumulated case and death rates for COVID-19 was to assess the temporal aspect of the COVID-19 spread by Brazilian regions for the ten epidemiological weeks, see Figure 4. This analysis allows us to obtain an important aspect regarding the spread of the disease in the country. The approach enables us to analyze whether the number of confirmed cases and deaths by COVID-19 in the last week has increased or decreased, according to the same interval as previous weeks in each region of the country, using a pattern recognition method. We can see that this approach can be taken as an analysis of the moving average, which is calculated by adding the number of cases from each of the previous seven days and dividing this result by seven. It is important to emphasize that it is not the cases or deaths during the week that necessarily enter the accounting but those registered in the system in this period.

This type of analysis is essential for the management of public measures to combat the pandemic. Therefore, the more precise and closer to our current reality these numbers are, the better-elaborated measures, and with this, more lives can be saved. In this sense, the SOM network allows us to verify trends in clusters, between epidemiological weeks, for novel and accumulated numbers of cases and deaths by COVID-19 per 100,000 inhabitants using an unconventional statistical method. For this, the network groups the data, so that the numbers of cases varied over time and not just the absolute values on each date. In this case, there is a difference in investigating absolute data or the variation trend.

During the epidemiological weeks, the SOM analysis showed a change in the spread of COVID-19 in the Brazilian regions, mainly represented by the North (N) and Northeast (NE) regions (Figure 4). In other words, in epidemiological weeks 20, 21, and 22, the rates of cases and deaths in these regions underwent significant behavior changes. These changes observed may represent the repercussion of the measures’ effects used in each region, which may come from less effective measures used to contain the spread of the virus or other non-controlling factors.

The SOM applied in this context does not evaluate the effects of the adopted measures, but it allows us to trace a general profile of the disease’s spread over time and verify which regions and epidemiological weeks had more significant dissimilarity. The weight maps also allow us to evaluate the curves of novel cases and deaths by COVID-19. It is possible to notice in Figure 4 that there is still an increase in the rates of novel cases and deaths according to the epidemiological weeks, which is represented by the formation of clusters in the upper right corner of the maps. Furthermore, another cluster represented all Brazilian regions in the first epidemiological weeks, lower left quadrant of the maps. If we were with falling rates, we would see that the epidemiological weeks 20, 21, and 22 would be classified in the lower left corner of the maps.

### 4.2. Spread of COVID-19 by Brazilian States

In the weight maps of Figure 6, we can show which states formed clusters. Among them, we highlight the cluster represented by the states of Amazonas (AM) and Pará (PA), with higher weights in the maps’ upper right corner. These states had the highest COVID-19 case and death rates recorded in the country. These two states were probably the most responsible for the North (N) region discrimination observed in Section 3.1 regarding the spread of COVID-19 according to Brazilian region. We can also verify that the states of Ceará (CE), Pará (PA), Pernambuco (PE), and Rio de Janeiro (RJ) formed a cluster in the lower left corner of the maps, which is mainly due to the intermediate rates of novel and accumulated deaths in these states. Therefore, these states’ death rates were higher than the other Brazilian states, except for the states of Amazonas (AM) and Pará (PA), see Figure 5 and Figure 6.

Figure 7 shows the numbers of novel and cumulative cases and deaths by COVID-19 in Brazilian states in each epidemiological week. According to the weight maps, the largest number of cases per 100,000 inhabitants is in the lower right corner, which is mainly represented by weeks 22 and 21 of Amapá (AP22), Amazonas (AM22), and Pará (PA22) states. The state of Ceará (CE22), Pernambuco (PE22), and Rio de Janeiro (RJ22) also showed high rates of novel and accumulated deaths in these epidemiological weeks. Compared to the other states, Goiás (GO), Minas Gerais (MG), Mato Grosso (MT), Mato Grosso do Sul (MS), Paraná (PR), Rio Grande do Sul (RS), and Santa Catarina (SC) have the lowest rates in the ten epidemiological weeks evaluated.

### 4.3. Spread of COVID-19 by Brazilian Cities

Evaluating the most densely populated areas in Brazil, represented by the metropolitan regions of São Paulo (SPA) and Rio de Janeiro (RIO), it is possible to observe that regions with large people agglomerations do not imply a higher rate of cases. For example, the metropolitan region of São Paulo, with around 20 million people, had fewer cases and deaths by COVID-19 per 100,000 inhabitants than the city of Boa Vista (BVA), capital of Roraima (RR), which has around 400,000 inhabitants, see Figure 8 and Figure 9.

Figure 10 represents the number of novel and accumulated cases and deaths of COVID-19 for the most populous cities of the country for each epidemiological week. The highest rates per 100,000 inhabitants of accumulated cases and deaths are in weeks 22 and 21 for the cities of Belém (BLM22 and BLM21), Manaus (MNS22 and MNS21), Fortaleza (FLA22 and FLA22), São Luís (SLS22 and SLS21), and Recife (RCE22 and RCE21), located in the upper right side of the maps.

Moreover, the weight maps for the novel cases and deaths had a change in the profile, where week 22 for São Luís (SLS22) and Macapá (MPA22) had the highest numbers of novel cases of COVID-19, and weeks 22, 21, and 20 for the cities of Boa Vista (BVA22, BVA21, and BVA20) and Belém (BLM22, BLM21, and BLM20) had the highest number of novel deaths among the cities evaluated.

### 4.4. Overview of the COVID-19 Spread in Brazil

In general, the SOM clustering ability was able to group cities, states, and regions spatially similar according to their coronavirus cases, which are represented by the same color scale on each map. Therefore, with this similarity, it is possible to benefit from using similar strategies to deal with the virus’ spread in these cities, states, and regions.

The spread of the novel coronavirus in Brazil does not have a standard behavior and varies between its regions, states, and cities. It is important to know that, in Brazil, the adopted policies to combat COVID-19 are the responsibility of each city or state. Lockdown, social isolation, closing non-essential services, public transport restrictions, and other measures have been adopted by authorities responsible for combating the virus [10].

The disease’s spread depends on other factors such as the number of hospital beds, health professionals, mass testing of the population, information level, and human development index in the region [29]. For this reason, it is necessary to insert these controllable factors, which for the most part depend on financial investments, in order to be able to state with greater certainty which measures have been more or less effective.

There is also evidence that COVID-19 proliferation is dependent on uncontrolled factors, such as the weather [30,31,32]. The location and the sizable territorial extension of Brazil give to the country different climate types, which vary considerably from region to region in the same season. Perhaps Brazil’s climate is a relevant factor in the diversity of COVID-19’s proliferation.

In the north of the country, the equatorial climate predominates, with rainfall throughout the year, with high relative humidity and average annual temperature between 25 and 27 °C, respectively. The northeast and central-west regions have a predominant tropical climate, with the semi-arid and tropical Atlantic climate in smaller proportions; however, most capitals in the northeast are in the region with a tropical Atlantic climate, with an average temperature between 18 and 28 °C. In the most densely populated region, the country’s southeast region dominates the tropical climate of altitude, with temperatures between 18 and 22 °C. Finally, the south region that predominates the subtropical climate is considered the lowest in the country with an average temperature of 18 °C [33].

## 5. Conclusions and Future Perspectives

The SOM clustering ability was able to spatially group similar cities, states, and regions according to their coronavirus cases, thus behaving similarly, so it is possible to benefit from using similar strategies to deal with the virus’ spread in these cities, states, and regions. The SOM applied in this context does not evaluate which measures applied have had effects to contain the disease’s spread in Brazil. However, the numbers of cases and deaths recorded by COVID-19 represent the repercussions of the measures effects used in each Brazilian region, state, or city to contain the virus’ spread.

Preliminary analyses using unsupervised neural networks of the Self-Organizing Map type showed that the spread of the novel coronavirus in Brazil does not have a standard behavior and varies between its regions, states, and cities. It was possible to verify that cities and states in the north and northeast of the country were the most affected by COVID-19. The results point out divergences with information disclosed by the media, which have considered only absolute values of cases and deaths for analysis. However, it is still not possible to point out why the north and northeast of the country were the most affected. In this sense, a more in-depth analysis is necessary, including controllable and uncontrollable factors, allowing the results to be explained with more security.

Future perspectives, studies including controllable and uncontrollable factors such as comorbidities, number of hospital beds, qualified professionals, human development index, and climate are already in progress. With data describing these factors, it will be possible to use Kohonen Maps’ clustering capability for the regions, states, or cities in Brazil with the same behavior and thus implement similar strategies to combat the coronavirus spread.

## Figures and Tables

**Table 1:** Percentage distribution of the Brazilian population by regions, states, and most populous cities and their respective abbreviations ^†^.

Brazil **BRA**/210,147,125	
North **N**/18,430,980 (9%)	
Acre **AC**/881,935 (0.4%)	Rio Branco **RBO**/407,319 (46.2%)
Amapá **AP**/845,731 (0.4%)	Macapá **MPA**/503,327 (59.5%)
Amazonas **AM**/4,144,597 (2.0%)	Manaus **MNS**/2,182,763 (52.7%)
Pará **PA**/8,602,865 (4.1%)	Belém **BLM**/1,492,745 (17.4%) Ananindeua **AIU**/530,598 (6.2%)
Rondônia **RO**/1,777,225 (0.8%)	Porto Velho **PVO**/529,544 (29.8%)
Roraima **RR**/605,761 (0.3%)	Boa Vista **BVA**/399,213 (65.9%)
Tocantins **TO**/1,572,866 (0.7%)	Palmas **PMJ**/299,127 (19.0%)
Northeast **NE**/57,071,654 (27%)	
Alagoas **AL**/3,337,357 (1.6%)	Maceió **MCO**/1,018,948 (30.5%)
Bahia **BA**/14,873,064 (7.1%)	Salvador **SDR**/2,872,347 (19.3%) Feira de Santana **FSA**/614,872 (4.1%) *
Ceará **CE**/9,132,078 (4.3%)	Fortaleza **FLA**/2,669,342 (29.2%)
Maranhão **MA**/7,075,181 (3.4%)	São Luís **SLS**/1,101,884 (15.6%)
Paraíba **PB**/4,018,127 (1.9%)	João Pessoa **JPA**/809,015 (20.1%)
Pernambuco **PE**/9,557,071 (4.5%)	Recife **RCE**/1,645,727 (17.2%) Jaboatão dos Guararapes **JBO**/702,298 (7.3%) *
Piauí **PI**/3,273,227 (1.6%)	Teresina **TSA**/864,845 (26.4%)
Rio Grande do Norte **RN**/3,506,853 (1.7%)	Natal **NTL**/884,122 (25.2%)
Sergipe **SE**/2,298,696 (1.1%)	Aracajú **AJU**/657,013 (28.6%)
Southeast **SE**/88,371,433 (42%)	
Espírito Santo **ES**/4,018,650 (1.9%)	Vitória **VTA**/362,097 (9.0%) Serra **SEA**/517,510 (12.9%) *
Minas Gerais **MG**/21,168,791 (10.1%)	Belo Horizonte **BHE**/2,512,070 (11.9%) Contagem **CEM**/663,855 (3.1%) * Juiz de Fora **JFA**/568,873 (2.7%) * Uberlândia **ULA**/691,305 (3.3%) *
Rio de Janeiro **RJ**/17,264,943 (8.2%)	Rio de Janeiro **RIO**/6,718,903 (38.9%) Belford Roxo **BFX**/510,906 (3.0%) * Campos dos Goytacazes **CPS**/507,548 (2.9%) * Duque de Caxias **DQX**/919,596 (5.3%) * Niterói **NRI**/513,584 (3.0%) * Nova Iguaçu **NIU**/821,128 (4.8%) * São Gonçalo **SGO**/1,084,839 (6.3%) *
São Paulo **SP**/45,919,049 (21.9%)	São Paulo **SPA**/12,252,023 (26.7%) Campinas **CAM**/1,204,073 (2.6%) * Guarulhos **GRH**/1,379,182 (3.0%) * Osasco **OSC**/698,418 (1.5%) * Ribeirão Preto **RPR**/703,293 (1.5%) * Santo André **SDE**/718,773 (1.6%) * São Bernardo do Campo **SBC**/838,936 (1.8%) * São José dos Campos **SJC**/721,944 (1.6%) * Sorocaba **SCB**/679,378 (1.5%) *
South **S**/29,975,985 (14%)	
Paraná **PR**/11,433,957 (5.4%)	Curitiba **CTA**/1,933,105 (16.9%) Londrina **LDA**/569,733 (5.0%) *
Rio Grande do Sul **RS**/11,377,239 (5.4%)	Porto Alegre **PAE**/1,483,771 (13.0%) Caxias do Sul **CSL**/510,906 (4.5%) *
Santa Catarina **SC**/7,164,788 (3.4%)	Florianópolis **FNS**/500,973 (7.0%) Joinville **JVE**/590,466 (8.2%) *
Central-West **CW**/16,297,074 (8%)	
Distrito Federal **DF**/3,015,268 (1.4%)	Brasília **BSA**/3,015,268 (100%)
Goiás **GO**/7,018,354 (3.3%)	Goiânia **GNA**/1,516,113 (21.6%) Aparecida de Goiânia **ACG**/578,179 (8.2%) *
Mato Grosso **MT**/3,484,466 (1.7%)	Campo Grande **CPE**/895,982 (32.2%)
Mato Grosso do Sul **MS**/2,778,986 (1.3%)	Cuiabá **CBA**/612,547 (17.6%)

^†^ Data reported by IBGE in 2019. * Non-capital cities with more than 500 thousand inhabitants.

**Table 2:** Geographic distribution dataset by regions, states, and most populous cities in Brazil for Self-Organizing Maps analysis (SOM).

ANN	Dataset ^†^	Distribution			Variables
**1**	20	Region	5 regions	Epidemiological week total sum	Novel and accumulated numbers of cases and deaths by COVID-19 per 100,000 inhabitants
**2**	200			Ten epidemiological weeks	
**3**	108	States	26 states plus the federal district	Epidemiological week total sum	
**4**	1080			Ten epidemiological weeks	
**5**	208	City	27 capital plus 25 other cities	Epidemiological week total sum	
**6**	2080			Ten epidemiological weeks	
	**3488**				

^†^ Values do not represent the case numbers of COVID-19; these values correspond to the dataset size representing the number of columns multiplied by the number of rows.

## Supplementary Materials

The following are available online at https://www.mdpi.com/1648-9144/57/3/235/s1, Figure S1: Temporal aspect of the COVID-19 spread in Brazilian cities per 100 thousand inhabitants. (Cumulative cases), Figure S2: Temporal aspect of the COVID-19 spread in Brazilian cities per 100 thousand inhabitants. (Novel cases), Figure S3: Temporal aspect of the COVID-19 spread in Brazilian cities per 100 thousand inhabitants. (Cumulative deaths), Figure S4: Temporal aspect of the COVID-19 spread in Brazilian cities per 100 thousand inhabitants. (Novel deaths).
