# Supplementary material for: The Spread of the COVID-19 Outbreak in Brazil: An Overview by Kohonen Self-Organizing Map Networks
Source: Medicina (Kaunas). 2021 Mar 3;57(3):235. doi: 10.3390/medicina57030235 (PMC7999572; doi:10.3390/medicina57030235)
Supplement: Supplementary file 1 [file medicina-57-00235-s001.pdf]

The Spread of the COVID-19 Outbreak in Brazil: An Overview by Kohonen Self-Organizing Map Networks

Diego Galvan [1,2,3,*], Luciane Effting [4], Hágata Cremasco [4] and Carlos Adam Conte-Junior [1,2,3]

1 COVID-19 Research Group, Center for Food Analysis (NAL), Technological Development Support Laboratory (LADETEC), Cidade Universitária, Rio de Janeiro, RJ 21941-598, Brazil; conte@iq.ufrj.br
2 Laboratory of Advanced Analysis in Biochemistry and Molecular Biology (LAABBM), Department of Biochemistry, Federal University of Rio de Janeiro (UFRJ), Cidade Universitária, Rio de Janeiro,
RJ 21941-909, Brazil
3 Nanotechnology Network, Carlos Chagas Filho Research Support Foundation of the State of Rio de Janeiro (FAPERJ), Rio de Janeiro, RJ 20020-000, Brazil
4 Chemistry Department, State University of Londrina (UEL), Londrina, PR 86057-970, Brazil; luciane.effting@uel.br (L.E.); hagata@uel.br (H.C.)
* Correspondence: diegogalvann@gmail.com or diegogalvann@iq.ufrj.br

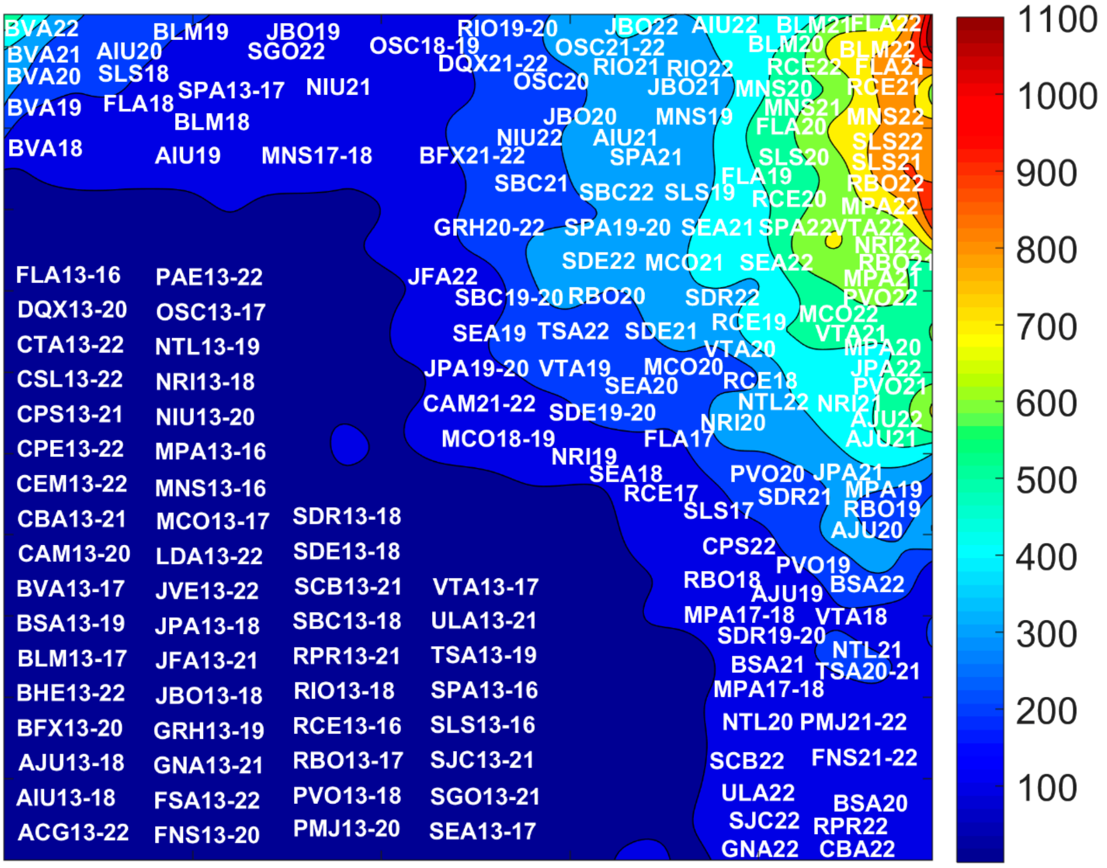

Figure S1 – Temporal aspect of the COVID-19 spread in Brazilian cities per 100 thousand inhabitants. **(Cumulative cases).**

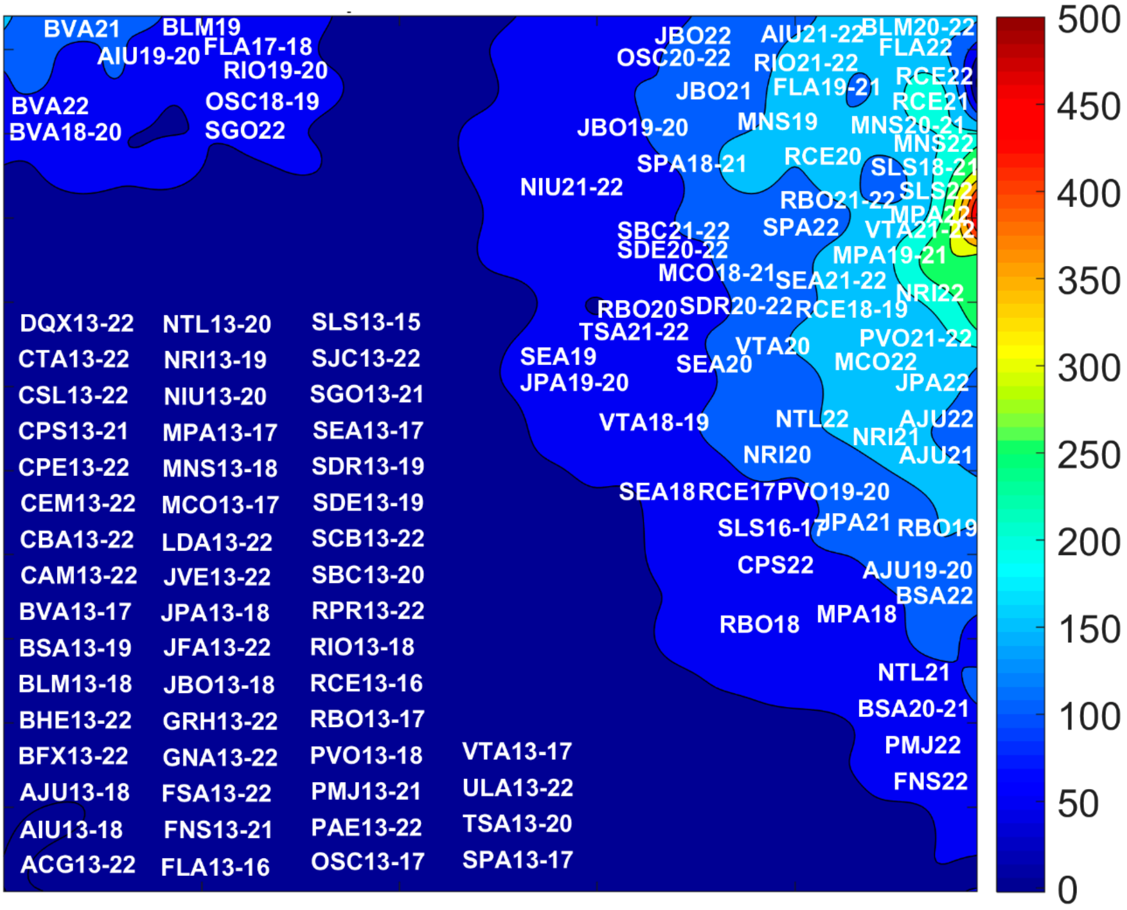

Figure S2 – Temporal aspect of the COVID-19 spread in Brazilian cities per 100 thousand inhabitants. **(Novel cases).**

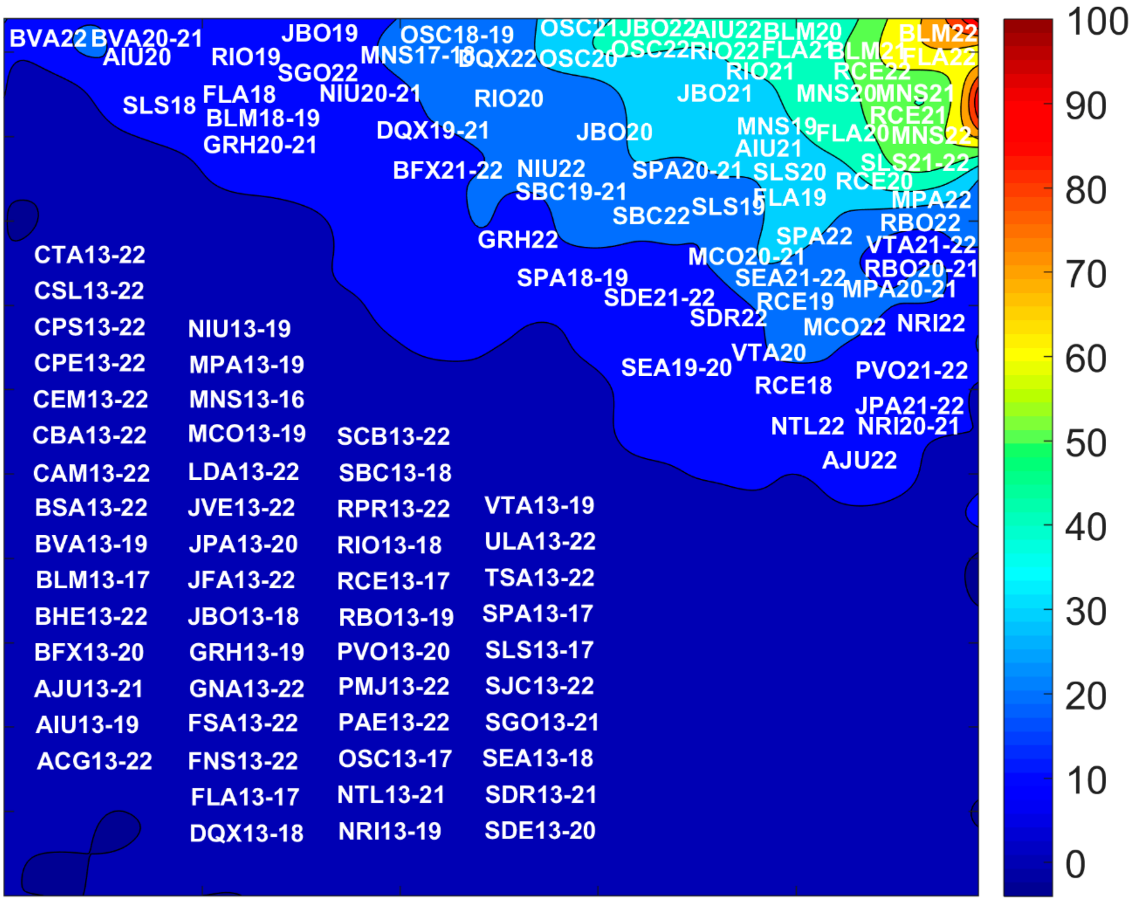

Figure S3 – Temporal aspect of the COVID-19 spread in Brazilian cities per 100 thousand inhabitants. **(Cumulative deaths).**

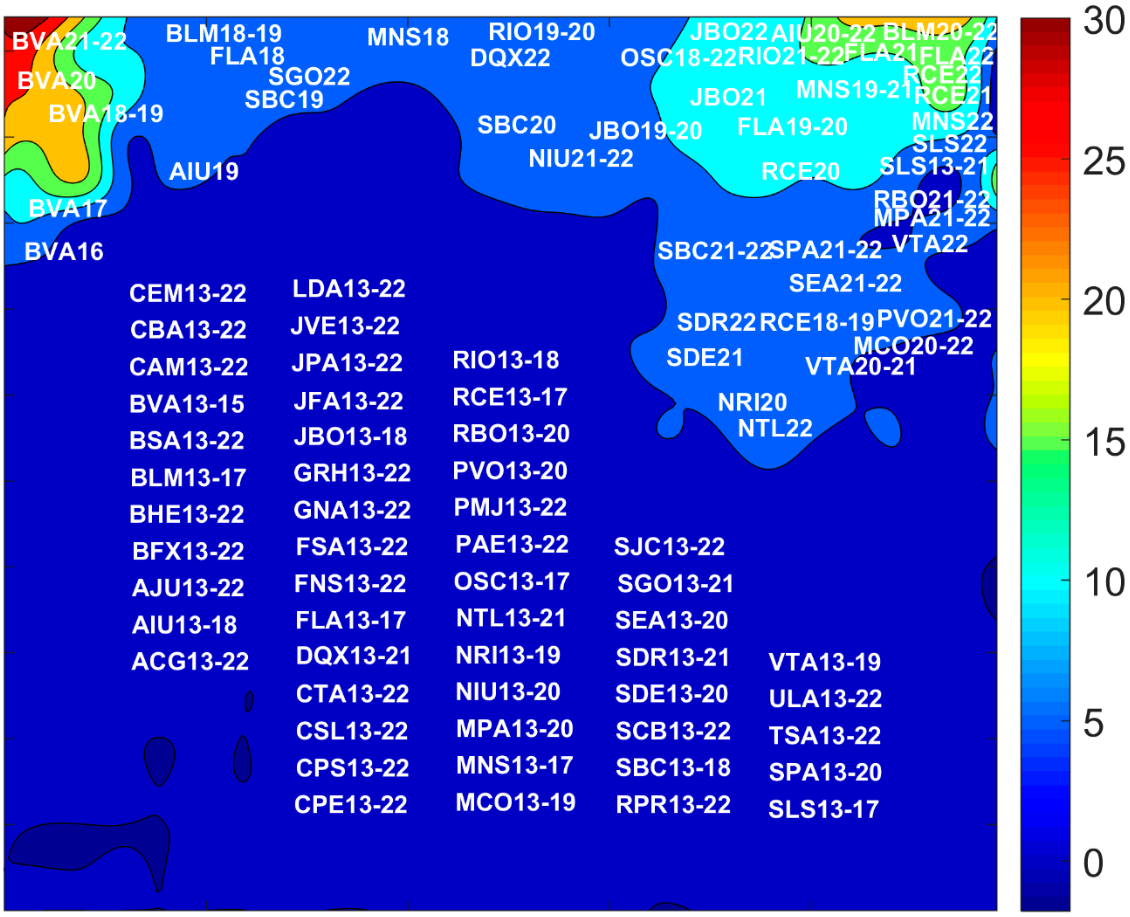

Figure S4 – Temporal aspect of the COVID-19 spread in Brazilian cities per 100 thousand inhabitants. **(Novel deaths).**
